# α-KG inhibits tumor growth of diffuse large B-cell lymphoma by inducing ROS and TP53-mediated ferroptosis

**DOI:** 10.1038/s41420-023-01475-1

**Published:** 2023-06-12

**Authors:** Yiqing Cai, Liemei Lv, Tiange Lu, Mengfei Ding, Zhuoya Yu, Xiaomin Chen, Xiangxiang Zhou, Xin Wang

**Affiliations:** 1 Department of Hematology, Shandong Provincial Hospital, Shandong University, Jinan, Shandong 250021 China; 2 Department of Hematology, Shandong Provincial Hospital Affiliated to Shandong First Medical University, Jinan, Shandong 250021 China; 3 Shandong Provincial Engineering Research Center of Lymphoma, Jinan, Shandong 250021 China; 4 Branch of National Clinical Research Center for Hematologic Diseases, Jinan, Shandong 250021 China; 5 National Clinical Research Center for Hematologic Diseases, the First Affiliated Hospital of Soochow University, Suzhou, 251006 China

**Keywords:** Lymphoma, Cancer therapy

## Abstract

Metabolic reprogramming is a hallmark of human malignancies. Dysregulation of glutamine metabolism is essential for tumorigenesis, microenvironment remodeling, and therapeutic resistance. Based on the untargeted metabolomics sequencing, we identified that the glutamine metabolic pathway was up-regulated in the serum of patients with primary DLBCL. High levels of glutamine were associated with inferior clinical outcomes, indicative of the prognostic value of glutamine in DLBCL. In contrast, the derivate of glutamine alpha-ketoglutarate (α-KG) was negatively correlated with the invasiveness features of DLBCL patients. Further, we found that treatment with the cell-permeable derivative of α-KG, known as DM-αKG, significantly suppressed tumor growth by inducing apoptosis and non-apoptotic cell death. Accumulation of a-KG promoted oxidative stress in double-hit lymphoma (DHL), which depended on malate dehydrogenase 1 (MDH1)-mediated 2-hydroxyglutarate (2-HG) conversion. High levels of reactive oxygen species (ROS) contributed to ferroptosis induction by promoting lipid peroxidation and TP53 activation. In particular, TP53 overexpression derived from oxidative DNA damage, further leading to the activation of ferroptosis-related pathways. Our study demonstrated the importance of glutamine metabolism in DLBCL progression and highlighted the potential application of α-KG as a novel therapeutic strategy for DHL patients.

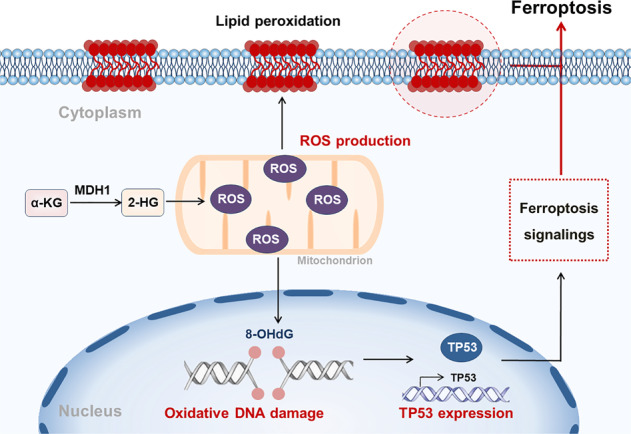

## Introduction

Diffuse large B-cell lymphoma (DLBCL) is the most common subtype of non-Hodgkin’s lymphoma (NHL), featured by heterogeneity, aggressiveness, and poor prognosis [1, 2]. With the emergence of anti-CD20 monoclonal antibodies, the survival rates for DLBCL patients have improved significantly. However, approximately 40% of cases progress to relapsed/refractory DLBCL [3]. Recently, molecular heterogeneity of DLBCL has been identified as a major factor interfering with clinical treatment responses [4], especially in double-hit lymphoma (DHL) and triple-hit lymphoma (THL) [5, 6]. Investigation of new therapeutic strategies is an urgent need to guide clinical practice.

As a prominent hallmark of cancer, metabolic reprogramming represents the phenotypic outcome and environmental influences on the molecular function of tumor cells [7]. Reorganization of tumor metabolism is characterized by increased assumption of various nutrients, including glutamine [8]. Glutamine is a vital metabolic fuel that is essential for ATP production and precursors biosynthesis [9]. Intracellular glutamine is converted into glutamate and alpha-ketoglutarate (α-KG) to support the tricarboxylic acid (TCA) cycle [10]. Glutamine metabolism also promotes nucleotides and protein biosynthesis to meet the demand for rapid tumor growth [11, 12]. In addition to energy supply, activation of glutamine metabolism contributes to counteracting the adverse effects of ammonia on tumor cells [13]. Therefore, glutamine metabolism plays an important role in tumor growth, microenvironment regulation, and drug resistance [14]. A recent study demonstrated that metabolic reprogramming-mediated glutaminolysis was associated with Bruton’s tyrosine kinase inhibitor (BTKi) resistance in mantle cell lymphoma (MCL) [15], suggesting the promise of targeting metabolism in hematological tumors.

As the major derivative of glutamine, a steady level of α-KG is essential for various biological processes [16, 17], while accumulation of α-KG conversely has tumor-suppressing effects. Elevated level of α-KG suppresses tumorigenesis by switching glucose metabolism [18, 19] and hinders tumor progression by driving tumor cell differentiation [20]. In addition, accumulated α-KG blocks tumor growth by inducing gasdermin C (GSDMC)-mediated pyroptosis [17], indicating that cell death induction by α-KG may be considered a potential strategy for cancer. Consistently, α-KG plays an important role in DLBCL, which depends on the functions of α-KG in promoting TCA process [21]. Since α-KG-induced cell death has yet to be described in DLBCL, investigating the mechanisms of α-KG will hope to provide novel therapeutic strategies for DLBCL patients.

Non-apoptotic cell death is evolving into a new cancer treatment strategy due to its influence on tumor progression and treatment response [22, 23]. As a novel form of non-apoptotic cell death, ferroptosis is derived from iron overload and lipid reactive oxygen species (ROS) accumulation [24]. In contrast to cell apoptosis, ferroptosis has specific morphological features, including shrinkage of mitochondria, reduction of mitochondrial cristae, and condensation of the internal membrane [25, 26]. Targeting ferroptosis is a promising approach to cancer therapy [27] since ferroptosis activation has crosstalk with metabolic reprogramming, anti-tumor immune regulation, and drug resistance [28, 29]. It is worth noting that ferroptosis inducers alone or combined with chemotherapy exhibit an inspiring anti-tumor efficacy [30]. Additionally, ferroptosis facilitates suppressing the progress of hematologic malignancies [31–33]. In particular, the accumulation of ROS and intracellular ferrous iron could enhance the sensitivity of DLBCL cells to ferroptosis [34, 35]. Herein, inducing ferroptosis is expected to be a new direction for the targeted therapy of DLBCL.

In this study, we demonstrated that glutamine metabolism was up-regulated in patients with primary DLBCL and related to adverse outcomes of patients. Accumulation of α-KG, an important metabolite of glutamine, conversely induced ferroptosis by promoting oxidative stress and activating TP53-mediated ferroptosis pathways in DHL cell lines. Our findings highlighted the potential of α-KG as a therapeutic agent and provided a novel option for DLBCL patients, especially those with DHL.

## Results

### Glutamine metabolism was up-regulated in DLBCL patients and associated with inferior outcomes

**Fig. 1:**
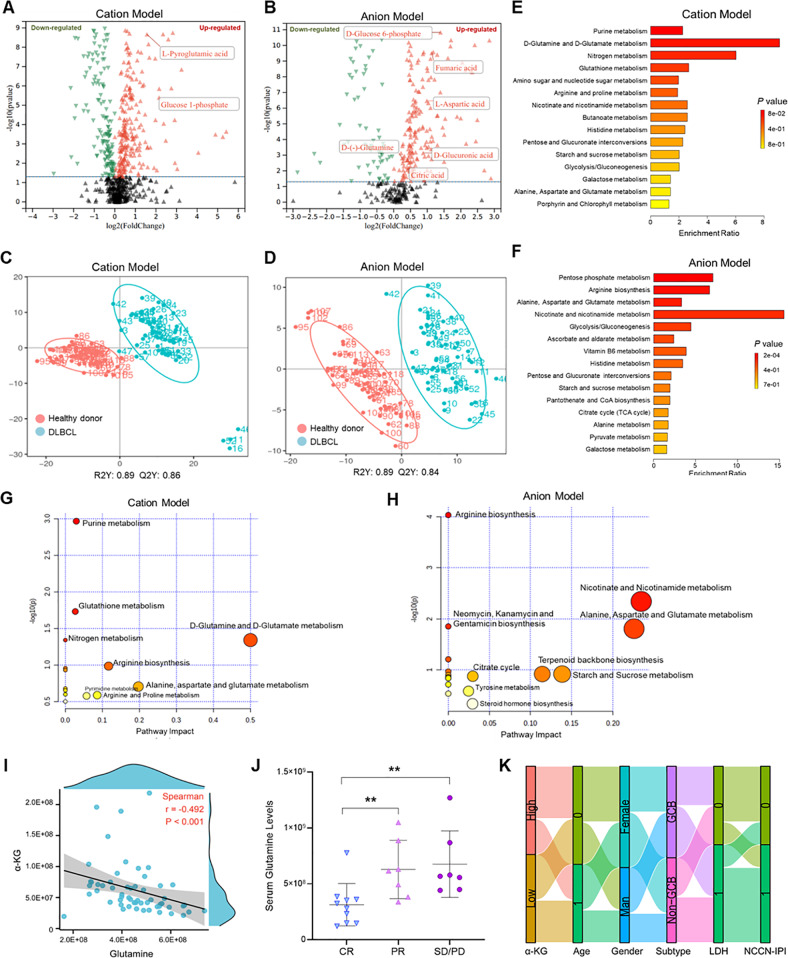
Glutamine metabolism was up-regulated and associated with poor prognosis of de novo DLBCL. **A**–**H** Metabolic patterns of DLBCL patients were analyzed based on serum untargeted metabolomics (DLBCL = 60, healthy donor = 60). Differentially expressed metabolites were identified in cation (**A**) and anion (**B**) models. The differentially expressed metabolites were used to construct PLS-DA. The cation PLS-DA score plot of DLBCL patients and healthy donors, which parameters were as follows: R2X = 0.89, Q2Y = 0.86 (**C**). The anion PLS-DA score plot of DLBCL patients and healthy donors, which parameters were as follows: R2X = 0.89, Q2Y = 0.84 (**D**). Functional enrichments of cation (**E**) and anion (**F**) differential metabolites were conducted in the KEGG database. Topological analysis was performed to identify the weight of differentially metabolic pathways in cation (**G**) and anion (**H**) models. **I** Correlation analysis between serum glutamine and α-KG (Spearman R = −0.492, *p* < 0.001). **J** Serum levels of glutamine in patients with different treatment responses after 6 cycles of treatments, including CR (*n* = 10), PR (*n* = 7), and SD/PD (*n* = 6). The data were compared using the two-tailed *t* test. **K** DLBCL patients were divided into two groups by the median concentration of α-KG. Alluvial diagram showing the changes of α-KG in age, gender (0 = male, 1 = female), subtype, LDH levels (0 ≤ 250 U/L, 1 > 250 U/L), and the NCCN-IPI score (0 = 0 ~ 3 scores, 1 = 4 ~ 6 scores).

To explore the metabolic profile of de novo DLBCL, we performed untargeted metabolomics of serum samples from 60 newly diagnosed DLBCL patients. Differential metabolites screening revealed that more than 280 metabolites were significantly changed in DLBCL patients compared with healthy donors (log2FC value > 1 and *p* value < 0.05; Fig. 1A, B). Among them, glucose-1-phosphate, D-glucose-6-phosphate, fumaric acid, citric acid, and D-glutamine were distinctly up-regulated, suggesting the elevation of glucose metabolism and TCA cycle in DLBCL patients (Fig. 1A, B). To profile the metabolic features of DLBCL patients, the differential metabolites were screened to construct the partial least squares discriminant analysis (PLS-DA). The satisfactory modeling and predictive ability of PLS-DA were shown both in the cation model (R2Y = 0.89, Q2 = 0.86) and the anion model (R2Y = 0.89, Q2 = 0.84), suggesting that the metabolic profile of DLBCL patients differed from that of healthy donors (Fig. 1C, D). Enrichment analysis in the Kyoto Encyclopedia of Genes and Genomes (KEGG) database was performed to investigate the differential metabolic pathways. As shown in Fig. 1E, differentially expressed cationic metabolites were mainly enriched in purine, nitrogen, and glutamate metabolisms. In addition, the enrichment of pentose phosphate, amino acid, nicotinamide, and glutamine metabolisms was revealed in the anion panel (Fig. 1F). Topological analysis further determined the importance of different metabolisms. The results showed that glutamine metabolism is the most important component of cationic and anion metabolic pathways (Fig. 1G, H). Taken together, our findings suggested the potential contribution of glutamine metabolism to the tumorigenesis of DLBCL.

To screen for metabolites that play an important role in glutamine metabolism, serum levels of glutamine-related metabolites were analyzed. Expression analysis demonstrated that glutamine, precursors (L-glutamic acid), and derivative (α-KG) were important in glutamine metabolism (Supplementary Fig. S1A). Notably, glutamine was negatively associated with α-KG, suggesting that α-KG might be used for glutamine synthesis in DLBCL patients (Fig. 1I). Since glutamine synthesis is essential for tumorigenesis, we then explored the clinical significance of glutamine metabolism by investigating the correlation between glutamine metabolites and patients’ clinical information. Our analysis revealed that high levels of serum glutamine were associated with adverse treatment response after 6 cycles of chemotherapy, indicating poor prognosis in patients with up-regulated glutamine (Fig. 1J). In contrast to glutamine, serum α-KG was reduced in DLBCL patients with high levels of lactate dehydrogenase (LDH) and National Comprehensive Cancer Network (NCCN)-international prognostic index (IPI) scores (Fig. 1K). To further assess the role of α-KG in predicting prognostic risk, α-KG levels were compared in patients with different LDH-ratio [36]. Unfortunately, α-KG levels showed no significant changes in the different groups (Supplementary Fig. S1B). These data support that glutamine metabolism plays an important role in the tumorigenesis of DLBCL.

### α-KG suppressed DLBCL cell growth by inducing cell death

**Fig. 2:**
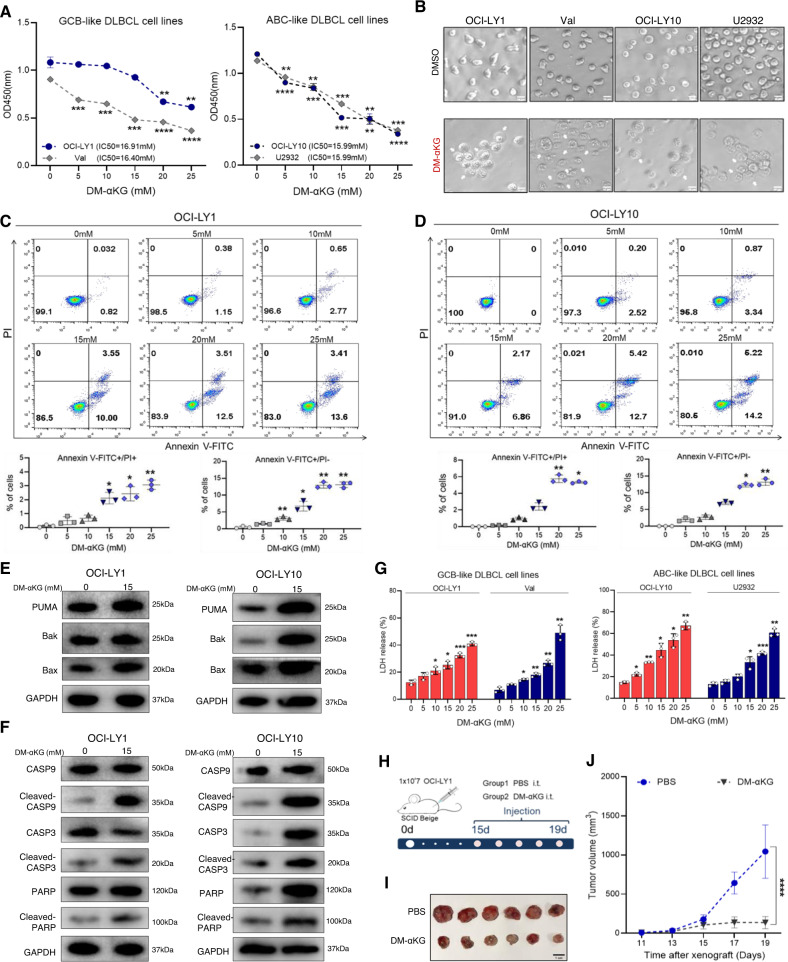
α-KG suppressed cell growth by inducing cell death in DLBCL. **A** GCB-like DLBCL cells (OCI-LY1, Val) and ABC-like DLBCL cells (OCI-LY10, U2932) were treated with 0.1% DMSO or DM-αKG (5, 10, 15, 20, 25 mM) for 24 h. CCK-8 assay was performed to detect cell proliferation. The IC50 of different cell lines were as follows: 16.91 mM (OCI-LY1), 16.40 mM (Val), and 15.99 mM (OCI-LY10 and U2932). **B** After treating with 0.1% DMSO or 15 mM DM-αKG for 24 h, morphology of OCI-LY1, Val, OCI-LY10, and U2932 cells were detected by light microscope (scale bar = 10 μm). **C**, **D** OCI-LY1 and OCI-LY10 cells were treated with 0.1% DMSO or DM-αKG (5, 10, 15, 20, 25 mM) for 24 h. Flow cytometry analysis showed the scatter plots, quantitative Annexin V-FITC^+^/PI^-^, and quantitative Annexin V-FITC^+^/PI^+^ in OCI-LY1 (**C**) and OCI-LY10 (**D**) cells. **E**, **F** OCI-LY1 and OCI-LY10 cells were treated with 0.1% DMSO or 15 mM DM-αKG for 24 h. Immunoblot analysis revealed the expression of apoptosis-related proteins, including PUMA, Bak, Bax (**E**), CASP9, cleaved-CASP9, CASP3, cleaved-CASP3, PARP, and cleaved-PARP (**F**). **G** OCI-LY1, Val, OCI-LY10, and U2932 cells were treated with 0.1% DMSO or DM-αKG (5, 10, 15, 20, 25 mM) for 24 h. Supernatant LDH levels were examined by the LDH release assay. **H–J** Four-week-old female SCID beige mice were subcutaneously injected with 1 × 10^7^ OCI-LY1 cells and randomized into 2 groups (*n* = 6/group). Vehicle (PBS, 100 μL per mouse) or DM-αKG (500 mg/kg) was intratumorally injected every day for 5 times (**H**). Images of tumor bodies were shown on day 20 (**I**, scale bar = 1 cm). Volume measurements demonstrated tumor growth at two-day intervals (**J**). All data were presented as means ± SD of 3 independent experiments. The data were analyzed by one-way ANOVA followed by Dunnett’s multiple comparison tests in (**A**, **C**, **D**, **G**), or two-way ANOVA with Sidak correction in (**J**). ^*^P < 0.05, ^**^P < 0.01, ^***^P < 0.001, ^****^P < 0.0001.

After revealing the correlation between the serum α-KG and clinical features, we examined the role of α-KG on DLBCL cell growth. To adjust the intracellular concentration of α-KG, DLBCL cell lines were treated with different concentrations of a cell-permeable derivative of α-KG, named dimethyl α-ketoglutarate (DM-αKG) [17]. As shown in Fig. 2A, DM-αKG treatment significantly suppressed cell proliferation of both germinal center B-cell (GCB)-like and activated B-cell (ABC)-like DLBCL cells. The effects of α-KG on cell death induction were then determined. Of note, DM-αKG exposure led to cellular shrinkage and cell membrane swelling in DLBCL cells, indicative of cell death induction (Fig. 2B). To verify the occurrence of cell death, we first detected the rate of apoptosis and the expression of apoptotic pathways. As expected, flow cytometry revealed a significant increase in apoptosis rates in the DM-αKG-treated group (Fig. 2C, D; Supplementary Fig. S2A, B). Consistently, immunoblot analysis showed that the protein levels of BCL-2 binding component 3 (BBC3, or PUMA), BCL-2-antagonist/killer 1 (Bak), and BCL-2-associated X protein (Bax) were increased by DM-αKG treatment, suggesting the activation of the mitochondrial apoptosis (Fig. 2E, Supplementary Fig. S2C). DM-αKG treatment also promoted the cleavage of caspase9 (CASP9), caspase3 (CASP3), and poly ADP-ribose polymerase (PARP) in DLBCL cells (Fig. 2F, Supplementary Fig. S2D). Given the increase in cell membrane swelling and Annexin V-FITC/PI double-positive cells, we further explored the generation of non-apoptotic cell death. Integrity of the cell membrane was determined by LDH release and expression. Interestingly, DM-αKG-treated cells were featured with high levels of supernatant LDH and reduced expression of LDHB (Fig. 2G, Supplementary Fig. S3A). These findings indicated that the supernatant LDH might be derived from the LDH previously stored in the cytoplasm, which referred to the disruption of the cell membrane integrity. The above results suggested that α-KG accumulation induced different forms of cell death, including apoptosis and non-apoptotic cell death.

To extend the functions of α-KG in vivo, we established a xenograft mouse model to determine the anti-tumor effects of DM-αKG (Fig. 2H). DM-αKG treatment dramatically decreased tumor size and depressed tumor growth in tumor-bearing mice (Fig. 2I, J). Collectively, our findings indicate that increasing α-KG inhibits cell growth by inducing cell death in DLBCL cells.

### α-KG induced redox imbalance in DHL cells

**Fig. 3:**
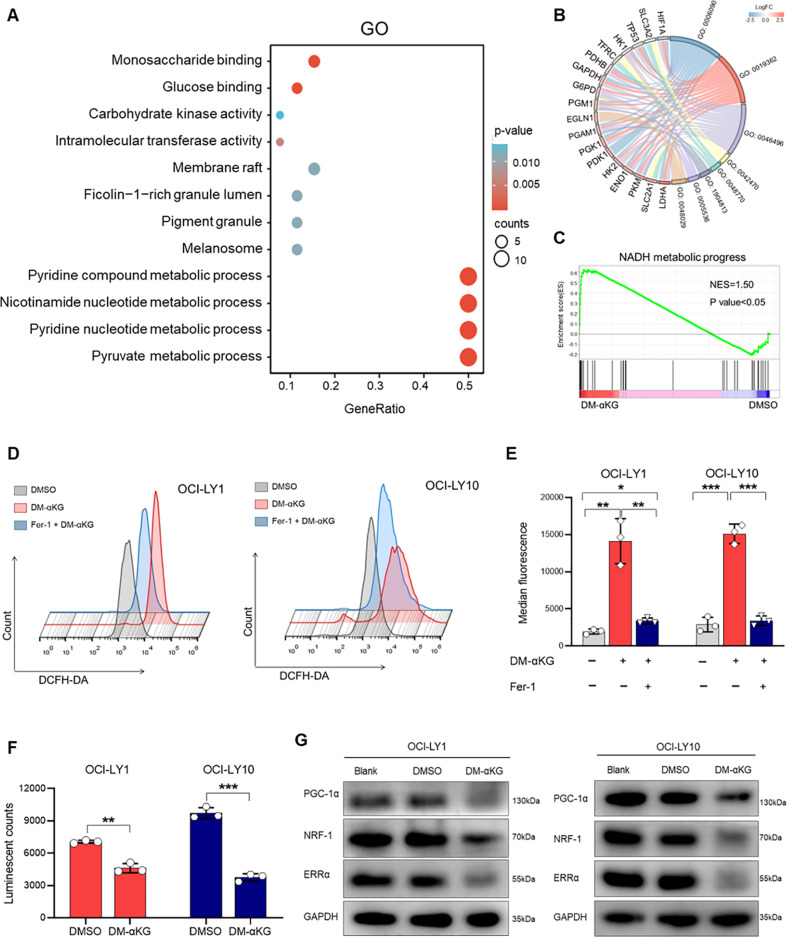
α-KG induced oxidative stress by promoting ROS production and ATP reduction in DHL. **A–C** OCI-LY1 and OCI-LY10 cells were treated with 15 mM DM-αKG for 24 h and performed RNA-seq (3 biological replicates for each group). Cells treated with 0.1% DMSO were used as control. Bubble plot profiled the most significantly differentially expressed GO terms (**A**). Contributing genes were displayed by a string diagram (**B**). GSEA plot of the NADH metabolic process (**C**, NES = 1.5, *p* < 0.05). **D**, **E** OCI-LY1 and OCI-LY10 cells were treated with different agents for 24 h: (1) 0.1% DMSO; (2) 15 mM DM-αKG; (3) 1 μM Fer-1 pre-treatment followed by 15 mM DM-αKG. DCFDA assay was performed to detect intracellular ROS levels, represented by flow cytometry plots (**D**) and relative fluorescence quantification (**E**). **F**, **G** OCI-LY1 and OCI-LY10 cells were treated with 0.1% DMSO or 15 mM DM-αKG for 24 h. Reagents containing D-luciferin and firefly luciferase were used to determine ATP levels, represented by the luminescence intensity (**F**). Immunoblot analysis revealed the protein levels of PCG-1α, NRF-1, and ERRα in DMSO- and DM-αKG-treated cells. Wild-type cells without drug treatment were used as the positive control (Blank) (**G**). All data were presented as means ± SD of 3 independent experiments. Immunoblot images in (**G**) were the representation of 3 independent experiments. The data were analyzed by two-tailed *t* test in (**E**) and (**F**). ^*^P < 0.05, ^**^P < 0.01, ^***^P < 0.001.

DHL is an aggressive form of DLBCL with an unmet treatment need [37]. A recent study highlighted that DNA damage response (DDR) was activated to enhance oxidative stress tolerance in DHL cells, facilitating resisting cell death [38]. Interestingly, DM-αKG treatment significantly induced cell death in DHL cell lines (OCI-LY1 and OCI-LY10). To investigate the underlining mechanisms of α-KG-induced cell death in DHL, RNA-sequencing (RNA-seq) was performed on DM-αKG-treated OCI-LY1 and OCI-LY10 cells. DeSeq2 analysis screened for differentially expressed genes (DEGs) in OCI-LY1 and OCI-LY10 cells and revealed 315 intersecting DEGs by Venn plot (Supplementary Fig. S4A). Subsequently, Gene Ontology (GO) analysis was conducted to investigate the functional alterations induced by DM-αKG. As shown in Fig. 3A, the pyridine-containing compound metabolic process, nicotinamide nucleotide metabolic process, pyridine nucleotide metabolic process, and glucose metabolism-related process were significantly enriched in DM-αKG-treated cells. Specific genes, such as hypoxia-inducible factor 1 subunit α (HIF1A), solute carrier family 3 member 2 (SLC3A2), and TP53, were the significant contributors to functional enrichment (Fig. 3B). Of note, pyridine nucleotides are essential in energy metabolism [39]. A typical example is nicotinamide adenine dinucleotide (NAD), which is responsible for redox homeostasis [40, 41]. Gene Set Enrichment Analysis (GSEA) consistently verified the elevation of NADH metabolic progress in DM-αKG-treated cells (Fig. 3C). Together with previous findings of reduced LDHB expression, the results suggested that increasing intracellular α-KG may contribute to redox imbalance in DHL cells.

### α-KG promoted ROS production and impaired ATP synthesis in DHL cells

The imbalance of the redox state promotes the production of peroxides, mainly manifested by the increase of ROS [42]. To determine whether α-KG could induce peroxidation in DLBCL, we used fluorescent probes to detect the intracellular levels of ROS in DM-αKG-treated cells. The results showed that DM-αKG exposure promoted a significant increase of 2’,7’-dichlorofluorescin diacetate (DCFDA) in DHL cells, representing the elevation of intracellular ROS (Fig. 3D, E). Moreover, α-KG-induced ROS could be reduced by a redox inhibitor Ferrostatin-1 (Fer-1) (Fig. 3D, E). These findings indicated that increasing α-KG induced a peroxidation reaction in DHL.

Next, we sought to investigate the effects of ROS on energy metabolism. ATP synthesis is known to be associated with ROS production [43]. GSEA revealed that ATP biosynthesis was down-regulated in DM-αKG-treated cells (Supplementary Fig. S5A). To further verify the inhibitory effect of energy production, we examined the expression levels of ATP and the mitochondrial regulatory pathway. As expected, DM-αKG treatment significantly reduced luminescent counts of ATP in DHL cells (Fig. 3F). Peroxisome proliferator-activated receptor-γ co-activator 1α (PGC-1α), nuclear respiratory factor 1 (NRF-1), and estrogen-related receptor alpha (ERRα) are the vital regulators for mitochondrial biogenesis [44, 45]. Consistent with reduced ATP, protein levels of PCG-1α, NRF-1, and ERRα were decreased in DM-αKG-treated cells (Fig. 3G). Consequently, our findings support that α-KG accumulation disrupts energy metabolism in DHL.

### Malate dehydrogenase 1 (MDH1) promoted ROS production by mediating the conversion of α-KG to 2-hydroxyglutarate (2-HG)

**Fig. 4:**
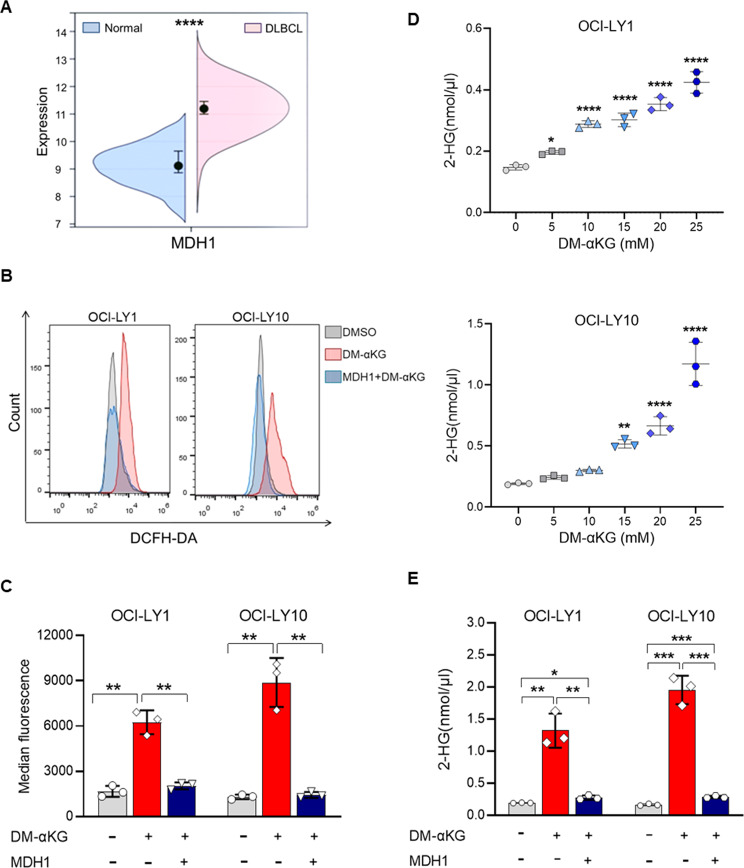
MDH1 promoted ROS production by mediating the conversion of α-KG to 2-HG. **A** The mRNA levels of MDH1 in DLBCL samples from the GEO database (GSE56315). **B**, **C** OCI-LY1 and OCI-LY10 cells were treated with different agents for 24 h: (1) 0.1% DMSO; (2) 15 mM DM-αKG; (3) 10 μM MDH1-IN-2 pre-treatment followed by 15 mM DM-αKG. DCFDA assay was applied to detect intracellular ROS levels, represented by flow cytometry plots (**B**) and relative fluorescence quantification (**C**). **D** OCI-LY1 and OCI-LY10 cells were treated with 0.1% DMSO or DM-αKG (5, 10, 15, 20, 25 mM) for 24 h. Supernatant 2-HG levels were examined by 2-HG assay. **E** OCI-LY1 and OCI-LY10 cells were treated with different agents for 24 h: (1) 0.1% DMSO; (2) 15 mM DM-αKG; (3) 10 μM MDH1-IN-2 pre-treatment followed by 15 mM DM-αKG. Supernatant 2-HG levels of drug-treated cells were detected by 2-HG assay. All data were presented as means ± SD of 3 independent experiments. The data were analyzed by two-tailed *t* test in (**A**, **C**, **E**), or one-way ANOVA followed by Dunnett’s multiple comparison test in (**D**). ^*^P < 0.05, ^**^P < 0.01, ^***^P < 0.001, ^****^P < 0.0001.

We then attempted to explore the origin of ROS in DM-αKG-treated DHL cells. Metabolic reprogramming of tumor cells might result in establishing the acidic tumor microenvironment (TME). We consistently found that glycolysis metabolism remained activated in DM-αKG-treated DHL cells, facilitating to acidic environment creation (Supplementary Fig. S5B). Interestingly, α-KG was found to promote ROS production by MDH1-mediated 2-HG conversion in an acidic environment [17]. DLBCL patients were featured by increased expression of MDH1, which suggested that DHL cells were qualified to transform α-KG (Fig. 4A). To determine whether MDH1 was involved in α-KG-induced ROS production, we detected ROS levels in DHL cells treated with MDH1 inhibitor or DM-αKG, respectively. Of note, MDH1 inhibition significantly reduced intracellular ROS induced by DM-αKG treatment (Fig. 4B, C). We further examined 2-HG levels to investigate the metabolite shift induced by MDH1. The results showed that 2-HG was increased by DM-αKG treatment in a dose-dependent manner (Fig. 4D). Besides, α-KG induced 2-HG was decreased by MDH1 inhibition (Fig. 4E). Therefore, ROS production is induced by the conversion of α-KG to 2-HG mediated by MDH1.

### α-KG-induced ferroptosis depended on the increase of lipid peroxidation

**Fig. 5:**
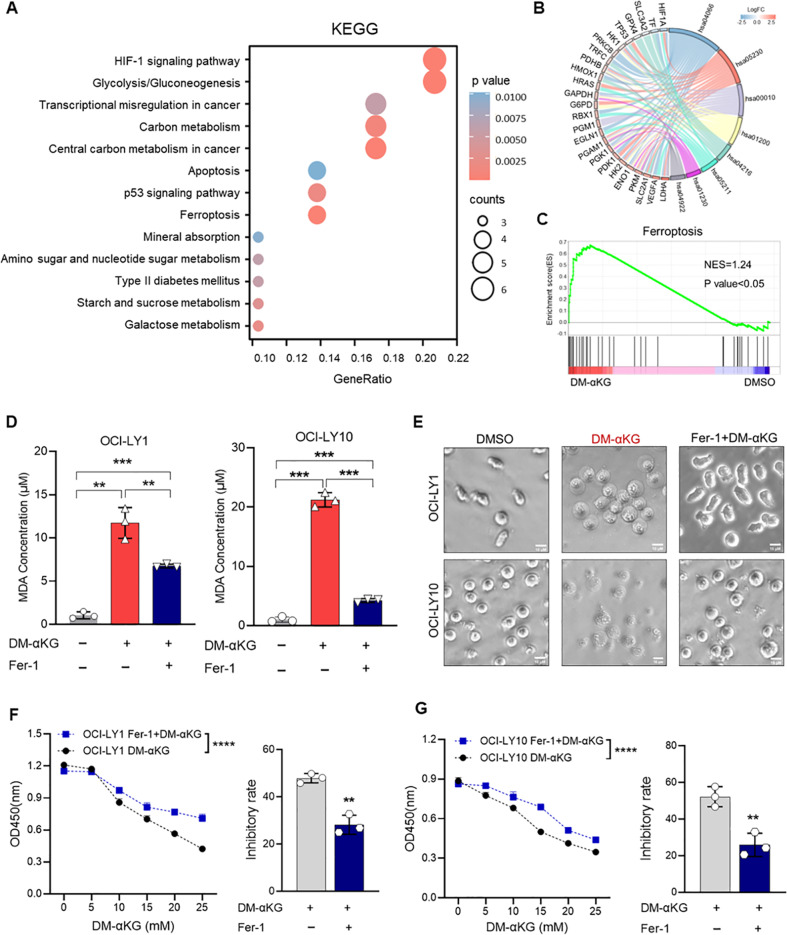
α-KG induced ferroptosis by promoting ROS-mediated lipid peroxidation in DHL. **A**–**C** Enrichment analysis of DM-αKG-treated OCI-LY1 and OCI-LY10 cells from RNA-seq. Cells treated with 0.1% DMSO were used as control. Bubble plot profiled the most significantly differentially expressed KEGG pathways (**A**). Contributing genes were displayed by a string diagram (**B**). GSEA plot of the Ferroptosis pathway (**C**, NES = 1.24, *p* < 0.05). **D**, **E** OCI-LY1 and OCI-LY10 cells were treated with different agents for 24 h: (1) 0.1% DMSO; (2) 15 mM DM-αKG; (3) 1 μM Fer-1 pre-treatment followed by 15 mM DM-αKG. Lipid peroxidation of drug-treated cells were detected by MDA assay (**D**). Cell morphology was detected by light microscope (**E**, scale bar =10 μm). The data were analyzed by a two-tailed *t* test. ^**^P < 0.01, ^***^P < 0.001. **F**, **G** Wild-type or 1 μM Fer-1 pre-treated OCI-LY1 and OCI-LY10 cells were incubated with 0.1% DMSO or DM-αKG (5, 10, 15, 20, 25 mM) for 24 h. Proliferation curves and inhibitory rates at 15 mM DM-αKG were determined by a CCK-8 assay. All data were presented as means ± SD of 3 independent experiments. Proliferation curves were analyzed by two-way ANOVA analysis with Sidak correction. Inhibitory rates were analyzed by two-tailed *t* test. ^**^P < 0.01, ^****^P < 0.0001.

After showing that increasing α-KG induced redox imbalance, we investigated whether α-KG-induced oxidative stress was associated with cell death induction. KEGG analysis revealed that the HIF-1 signaling pathway, p53 signaling pathway, and ferroptosis were significantly enriched in DM-αKG-treated DHL cells, of which contributing genes included SLC3A2, TP53, transferrin (TF), glutathione peroxidase 4 (GPX4), and heme oxygenase 1 (HMOX1) (Fig. 5A, B). GSEA consistently profiled an increased expression of ferroptosis in DM-αKG-treated cells (Fig. 5C), suggesting that α-KG-induced non-apoptotic cell death referred to ferroptosis. Ferroptosis was regarded as a cellular response to oxidative stress, which depended on lipid peroxidation [46, 47]. To verify the appearance of ferroptosis, we examined malonic dialdehyde (MDA) levels in DHL cells with DM-αKG treatment. As a result, MDA levels were significantly increased by DM-αKG and decreased by Fer-1 (Fig. 5D). We further examined the alterations in cell morphology and proliferative activity to explore whether suppressing ferroptosis could rescue DM-αKG-induced proliferation inhibition. As expected, Fer-1 treatment could reduce cell membrane swelling and proliferation inhibition in DM-αKG-treated cells (Fig. 5E–G). Together, these data identify α-KG-induced ferroptosis in DHL.

### Oxidative DNA damage induced TP53 expression to regulate α-KG-induced ferroptosis

**Fig. 6:**
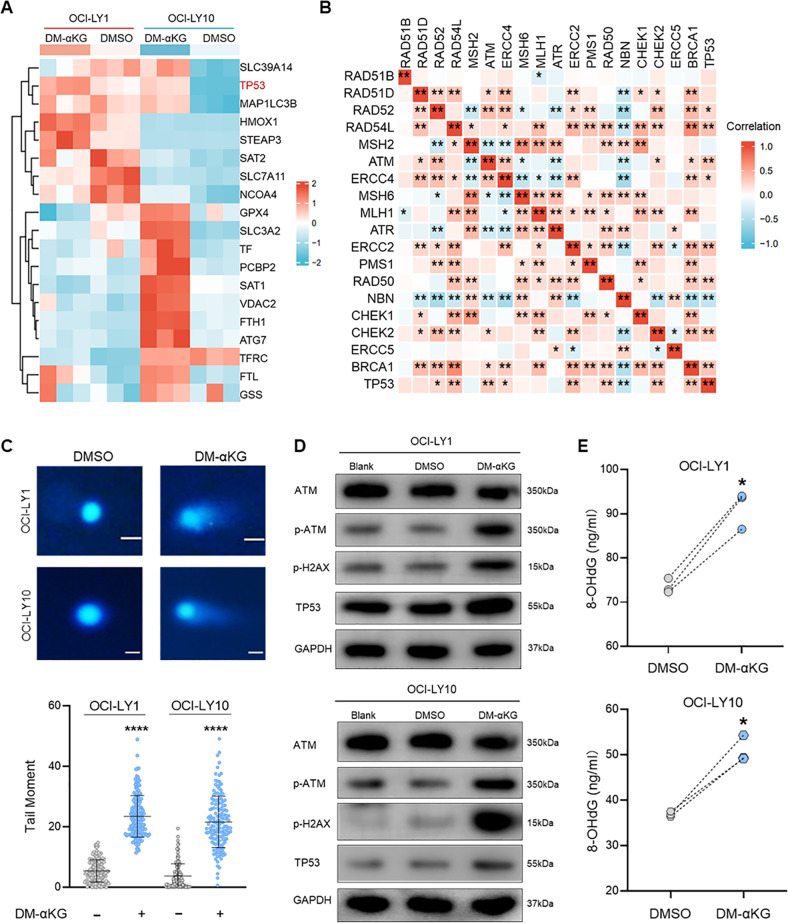
α-KG promoted oxidative DNA damage and TP53 expression to mediate ferroptosis. **A** Heatmap analysis of ferroptosis pathway in RNA-seq. **B** Correlation analysis of TP53 and DNA damage-related genes in DLBCL samples from the GEO database (GSE56315). **C–E** OCI-LY1 and OCI-LY10 cells were treated with 0.1% DMSO or 15 mM DM-αKG for 24 h. DNA fragments were examined by neutral comet assay, which were stained with DAPI (upper panel). DNA damage was determined by tail moments calculation, where 50 cells were counted in each individual experiment (lower panel) (**C**). Protein levels of ATM, p-ATM, p-H2AX, and TP53 were examined by immunoblot analysis, where wild-type cells without drug treatment were used as the negative control (Blank) (**D**). Oxidative DNA damage was determined by the 8-OHdG detection assay (**E**). Immunoblot images in (**D**) were the representation of 3 independent experiments. All data were presented as means ± SD of 3 independent experiments. The data were analyzed by two-tailed *t* test in (**C**) and (**E**). ^*^P < 0.05, ^****^P < 0.0001.

To further understand the regulatory mechanisms in α-KG-induced ferroptosis, we analyzed DEGs involved in the ferroptosis pathway. Heatmap analysis revealed that ferroptosis-related genes were differentially expressed in OCI-LY1 and OCI-LY10 cells (Fig. 6A). Among them, TP53 was significantly increased by DM-αKG treatment, indicating the potential involvement of TP53 in α-KG-induced ferroptosis (Fig. 6A). Correlation analysis was then conducted to investigate the associated factors which were involved in the regulation of TP53 in DLBCL. As demonstrated in Fig. 6B, TP53 expression was associated with elevated DDR and DNA damage repair. Therefore, we hypothesized that α-KG accumulation induced DNA damage to regulate TP53 expression in DHL cells. This hypothesis was supported by the results of comet assay and WB. Detection of DNA strand breakage (DSB) by the comet assay revealed a significantly increased tail moment after DM-αKG treatment, verifying the induction of DNA damage (Fig. 6C). Consistently, high levels of DNA damage biomarkers, including phosphorylated-Ataxia Telangiectasia Mutated (p-ATM) and phosphorylated-H2AX (p-H2AX), were detected in DM-αKG-treated cells, accompanied by an increase of TP53 (Fig. 6D). These results suggested that TP53 overexpression resulted from α-KG-induced DNA damage in DHL cells. Previous studies have illustrated that oxidative stress is a vital source of DNA damage [48, 49]. Given the function of α-KG in peroxidation induction, we further investigated whether DNA damage resulted from high levels of ROS. As shown in Fig. 6E, the release of 8-hydroxydeoxyguanosine (8-OHdG), an indicator of oxidative DNA damage, was significantly increased in DM-αKG-treated cells. The results suggest that α-KG-induced ROS promotes TP53 expression by regulating oxidative DNA damage in DHL.

## Discussion

**Fig. 7:**
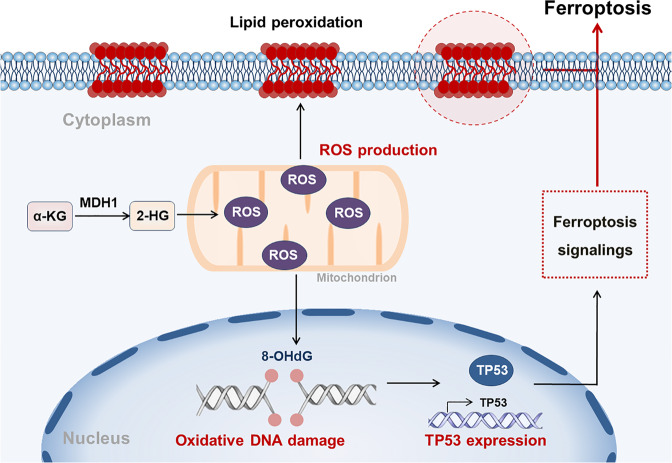
A working model of α-KG-induced ferroptosis in DHL. Intracellular α-KG was converted to 2-HG by MDH1, further promoting ROS production. ROS accumulation promoted lipid membrane peroxidation and initiated oxidative DNA damage. TP53 overexpression derived from oxidative DNA damage participated in regulating the ferroptosis pathways. TP53-mediated ferroptosis pathways, together with lipid peroxidation, further led to the generation of ferroptosis.

Here, we report that glutamine metabolism was up-regulated in DLBCL patients, which was characterized by elevated glutamine and decreased α-KG. Accumulative α-KG was converted to 2-HG by MDH1, which led to the production of ROS in DHL cells (Fig. 7). Increasing ROS further induced ferroptosis by promoting lipid peroxidation and DNA damage-mediated TP53 expression (Fig. 7). Based on the functions of α-KG in ferroptosis induction, DM-αKG treatment was found to inhibit tumor growth of DLBCL, especially cells with double-hit features. Our findings identify the novel mechanisms for ferroptosis via metabolite regulation in DLBCL, hoping to provide a new therapeutic strategy for DLBCL patients.

Metabolic reprogramming is essential for maintaining the biological functions of tumor cells and immune cells [7, 50]. As a kind of aggressive B-cell lymphoma, DLBCL is characterized by metabolic heterogeneity that is associated with drug resistance and immune microenvironment remodeling [51–53]. Understanding the metabolic features of DLBCL patients is vital for addressing the unmet medical need. Our work demonstrated an increased glutamine metabolism in DLBCL patients, consistent with the findings that glutamine metabolism is up-regulated to meet the demands of tumorigenesis [54–56]. In addition, the critical metabolite of glutamine metabolism, known as glutamine, was associated with adverse outcomes, indicating the prognostic value of glutamine in DLBCL. Given the limitations of single-center samples in representing the disease characteristics, a large sample validation will be conducted in future studies.

The importance of glutamine metabolism in DLBCL refers to its contributions to the TCA cycle, in which the glutamine metabolite α-KG is used as specific fuel [21]. As a highly aggressive lymphoma, α-KG of DLBCL patients was maintained at a low level, indicating an active energy metabolism in DLBCL cells. Since tumor invasiveness was associated with increased serum LDH (ratio>1) [36], serum α-KG was negatively correlated with serum LDH in DLBCL patients. However, α-KG levels showed no significant changes in different LDH-ratio groups, which may be limited by the insufficiency of patient samples. Recent studies demonstrated that the accumulation of intracellular α-KG leads to tumor-suppressing effects [57]. On one hand, α-KG accumulation disrupts glutamine anaplerosis by reversing glutamine-mediated nitrogen metabolism [54, 58]. On the other hand, α-KG is associated with induction of cell death, including pyroptosis and ferroptosis. Accumulative α-KG resulted in oxidative stress, which affected the redox state of cancer cells and ferroptosis induction [59, 60]. Specifically, α-KG induced ferroptosis by altering the redox state in OCI-LY1 and OCI-LY10 cell lines which harbor c-MYC rearrangement/amplification and BCL-2 rearrangement [61]. MYC hyperactivation could enhance the tolerance to oxidative stress and DNA damage, leading to poor prognosis in DHL patients [38]. Interestingly, α-KG-induced ROS significantly disrupted redox homeostasis, which contributed to overcoming MYC-mediated oxidative stress and oxidative DNA damage. Therefore, α-KG might be considered a promising therapeutic strategy for DHL patients.

As an oxidative, iron-dependent form of non-apoptotic cell death, ferroptosis locates at the intersection of metabolic modulation and signaling transduction [62]. Here, we demonstrated that α-KG-induced ferroptosis relied on lipid peroxidation and TP53-mediated signalings. Previous studies illustrate the critical role of TP53 in predicting outcomes of DLBCL patients [63]. Notably, our work highlighted an important mechanism by which TP53 regulated ferroptosis pathways in DHL cells. Interestingly, differentially activated ferroptosis pathways were detected in DLBCL cells with different TP53 mutation states and genetic features. A previous study illustrated that the steady-state levels of TP53 were higher than that of TP53^WT^ OCI-LY10 cells, even though OCI-LY1 cells were featured with mutant TP53 [64]. Given the stable expression of TP53 in OCI-LY1 cells, α-KG-induced ferroptosis might depend on the TP53-mediated reduction of solute carrier family 7 member 11 (SLC7A11) and GPX4 [65]. In contrast to OCI-LY1 cells, ABC-like OCI-LY10 cells constitutively activated the nuclear factor kappa-B (NF-κB) pathway to resist oxidative stress [66, 67]. Notably, NF-κB activation was associated with iron metabolism, indicating the potential of GPX4-independent ferroptosis in OCI-LY10 cells [68, 69]. Future studies will focus on revealing regulatory mechanisms of ferroptosis pathways in different subtypes of DLBCL.

Another vital function of α-KG refers to the induction of DNA damage. Evidence shows cellular DNA damage regulates cell proliferation and death [70, 71]. In particular, the invasive manifestations and chemoresistance of DHL are associated with the hyperactivation of DDR in DHL cells [38]. Accumulation of intracellular α-KG exacerbated oxidative stress and DNA damage to break the protective effects of DDR and eventually induced cell death. Therefore, our results indicate that α-KG treatment may improve the sensitivity of DDR-targeted therapy in DHL, which is expected to be an effective therapeutic option for DHL patients.

In summary, our study demonstrated the importance of glutamine metabolism in the tumorigenesis of DLBCL and the mechanisms of α-KG in ferroptosis induction. α-KG-based therapy may be a promising strategy for DLBCL, particularly for DHL patients.

## Materials and methods

### Peripheral serum samples and cell lines

A total of 120 peripheral serum samples, which included 60 patients with initially diagnosed DLBCL and 60 healthy volunteers, were collected from 2020 to 2022 in Shandong Provincial Hospital (Table 1). Serum samples were stored at −80 °C until analysis. The diagnostic criteria were based on the guidelines of the 2017 World Health Organization (WHO) [72]. 53 enrolled patients received treatment, of which 35 patients completed effect evaluation. Therapeutic regimens were shown in Supplementary Table S1. Clinical treatment responses were evaluated after every 3 cycles of treatment, which was based on positron emission tomography/computed tomography (PET/CT) results. Human DLBCL cell lines OCI-LY1, OCI-LY10, Val, and U2932 were grown in Iscove’s modified Dulbecco’s medium (IMDM) (Gibco, CA, USA) supplemented with 10% heat-inactivated fetal bovine serum (Gibco). Cells were maintained in a 37 °C, 5% CO_2_ humidified cell incubator. All cells were periodically examined for 15 mycoplasma infection and STR (Short Tandem Repeat).

**Table 1:** Clinical information of DLBCL patients (*n* = 60) enrolled in untargeted metabolomics.

Characteristics	Numbers
Age (years)	
≤60	29
>60	31
Gender	
Male	27
Female	33
Subtype	
GCB	30
ABC	30
Ann Arbor stage	
I or II	15
III or IV	45
NCCN-IPI score	
0–3	35
4–6	25
Serum LDH levels	
Normal (LDH-ratio ≤ 1)	24
Upper limit of normal (LDH-ratio > 1)	31
ENI	
Involved site (≤1)	7
Involved site (> 1)	44
Genetic features	
TP53 loss	7
TP53 mutant	1
BCL-6 rearrangement	8
MYC/BCL-2 rearrangement	1
Expression features	
Double expression	16
Treatment response to 6 cycles of treatments	
CR	10
PR	7
SD/PD	6

*GCB* germinal center B-cell, *ABC* activated B-cell, *NCCN-IPI* National Comprehensive Cancer Network-International Prognostic Index, *LDH* lactate dehydrogenase, *ENI* extranodal involvement, *DHL* double-hit lymphoma, *CR* complete remission, *PR* partial remission, *NR* non-remission, *SD* stable disease, *PD* progressive disease.

### Untargeted metabolomics

The untargeted metabolomics profiling was performed by Novogene Co., Ltd. (Beijing, China). Briefly, the peripheral serum samples from 60 DLBCL patients were resuspended with 80% methanol and diluted with LC-MS grade water. After centrifugation, the supernatant was injected into the LC-MS/MS system analysis that consisted of a Vanquish UHPLC system (ThermoFisher, Germany) coupled with an Orbitrap Q Exactive^TM^ HF-X mass spectrometer (Thermo Fisher). Both positive and negative polarity modes were operated. The raw data files were processed by peak alignment, peak picking, quantitation analysis, and data normalization to predict the molecular formula, followed by the annotation of metabolites. Principal components analysis (PCA) and PLS-DA were performed on the metaX. Differentially expressed metabolites, enrichment, and topology analysis were conducted by the R package.

### RNA-seq

To explore the underlying mechanisms of α-KG, the total RNA of DM-αKG-treated OCI-LY1 and OCI-LY10 cells was extracted by TRIzol reagent (Invitrogen, 15596026, CA, USA) and performed RNA-seq experiments (Novogene). Sequencing libraries were generated from purified mRNA by the NEBNext UltraTM RNA Library Prep Kit for Illumina (NEB, MA, USA) and clustered on the cBot cluster generation system. Subsequently, the prepared library was sequenced on the Illumina HiSeq platform and produced 150 bp paired-end reads. HTSeq v0.6.0 was applied to identify the reads and fragments per kilobase of transcript per million mapped reads (FPKM). GO, KEGG, GSEA, and correlation analysis were performed by using the R package.

### ROS detection and apoptosis assay

Intracellular ROS levels and cell apoptosis were measured by flow cytometry analysis. For ROS detection, DLBCL cells resuspended with serum-free IMDM were incubated with 10 mm cell-permeated DCFDA (Abcam, ab113851, MA, USA) for 15 min at 37°C. For apoptosis assay, cells were stained with Annexin V-FITC and PI for 15 min (BD Biosciences, 556547, MA, USA). Fluorescence was then measured by flow cytometry on Navios Flow Cytometer (Beckman Coulter, CA, USA). Images were processed by FlowJo software (v10.8.1, BD, USA).

### Lipid peroxidation assay

Cells were harvested and lysed in radioimmunoprecipitation (RIPA) buffer (Beyotime, P0013B, Shanghai, China). The protein lysates were diluted to appropriate concentrations and processed MDA assay using the lipid peroxidation MDA assay kit (Beyotimes, S0131S). The MDA levels were detected by a Multiskan GO microplate reader (Thermo Scientific, Rockford, IL, USA) at 532 nm.

### Luciferase-based ATP assay

A luminescent ATP detection assay kit (Abcam, ab113849) was used to measure ATP production. Briefly, cells were lysed and stabilized by detergent solution, further incubating with a reaction mixture containing D-luciferin and firefly luciferase reagent. The luminescence of luciferase activity was recorded by the Ultra-Sensitive Microplate Chemiluminescence (Berthold, LB 960, Germany).

### Oxidative DNA damage detection and 2-HG assay

Oxidative DNA damage was measured by an 8-OHdG competitive ELISA kit (Elabsciences, E-EL-0028, Beijing, China) according to the manufacturer’s protocol. The 2-HG assay was performed as described in the kit protocol (Abcam, ab211070). The optical density of the supernatant was detected by a Multiskan GO microplate reader (Thermo Scientific) at 450 nm.

### Neutral comet assay

The neutral comet assay was performed according to the manufacturer’s guidelines (Trevigen, Maryland, USA). DNA fragments were stained by DAPI (Solarbio, S2110, Beijing, China) and captured by the Olympus BX51 fluorescence microscope. The Open Comet software (Cambridge, USA) was used to analyze tail moments, of which 50 individual cells were calculated in each experiment.

### LDH detection assay and morphology observation

Serum LDH levels of the cells were measured by the CytoTox 96 non-radioactive cytotoxicity assay kit (Promega, G1780, Madison, USA). For morphology observation, cells were plated into 6-well plates (1 × 10^5^ cells/well) and observed by OLYMPUS CKX41 inverted microscope with ×200 objective. Each well was observed in 3 different fields and the respective image was processed by Image J software (National Institutes of Health, USA).

### Cell proliferation assay and reagents

Cell proliferation rates and viability were measured by the cell counting kit-8 (CCK-8) assay (Dojindo, CK04, MD, USA). Cells were seeded in 96-well plates at 1 × 10^4^ cells/well, harvested, and stained with 10 μl CCK-8/well, of which optical density was detected at 450 nm by Multiskan GO Microplate Reader (Thermo Scientific). DM-αKG was from Sigma Aldrich (Cat# 349631, Darmstadt, Germany). Fer-1 was from Selleckchem (S7243, TX, USA). MDH1 inhibitor MDH1-IN-2 was from MCE (HY-147791, New Jersey, USA).

### WB

WB was performed as previously described [73]. Cells were lysed in RIPA buffer (Beyotime, P0013B) supplemented with protease inhibitors. 40 μg of total proteins were loaded in the SDS-PAGE (Bio‐Rad, California, USA) and transferred to nitrocellulose membranes. According to the molecular weight of the target protein, the membranes were blocked with 10% skimmed milk and further incubated with primary antibodies at 4°C overnight. The membranes were re-probed with secondary antibodies for 1 h at room temperature. Antibodies were listed in Supplementary Table S2.

### In vivo mice models

The animal experiments were conducted by guidelines approved by Shandong University Hospital’s Institutional Animal Care and Research Advisory Committee. Four-week-old female severe combined immunodeficiency (SCID) beige mice of specific pathogen-free (SPF) class were purchased from the Vital River Laboratory Animal Technology Co., Ltd. (Beijing, China). To construct xenograft DLBCL models, 1 × 10^7^ wild-type OCI-LY1 cells were suspended in 200 μL PBS and subcutaneously injected into the right lower limb of mice. When tumors reached 100 mm^3^, mice were blindly assigned to two groups (*n* = 6/group) and injected with either vehicle (PBS, 100 μL per mouse) or DM-αKG (500 mg/kg), administered via intratumoral (i.t.) injections once per day for 5 days. Tumor volume was measured every 2 days and calculated as *V* = (*l* × *w*^2^) × 0.5. Mice were sacrificed until any one of several criteria were met, which included severe lethargy, more than 10% body-weight loss, and approximately 2 cm of tumor diameter.

### Statistical analysis

Each experiment was carried out at least 3 times, every individual experiment containing 3 replicates. A minimum of three independent experiments were displayed with mean ± standard deviation (SD). SPSS Statistics version 20.0 and GraphPad Prism software (v8.0a, La Jolla, CA, USA) were applied to the statistical analysis. The statistical significance between the two groups was determined by the unpaired two-tailed *t* test with assumed normal distribution. Three or more groups were analyzed by Welch’s one-way ANOVA analysis if normality tests passed, followed by Tukey’s multiple comparison tests. If normality tests failed, the Kruskal-Wallis test was performed with Dunnett’s T3 tests. Proliferation curves were measured by two-way ANOVA analysis with Sidak correction. Significant statistical significance was defined as *P* value < 0.05. ^*^P < 0.05, ^**^P < 0.01, ^***^P < 0.001, and ^****^P < 0.0001.

## Supplementary information

Supplementary Figures

Supplementary Tables

## Data Availability

The correlation analysis of TP53 and the expression analysis of MDH1 in DLBCL cohort were retrieved from the GEO database (https://www.ncbi.nlm.nih.gov/geo/query/acc.cgi?acc=GSE56315). Any additional data used and/or analyzed during the current study are available from the corresponding authors on reasonable request.
