# Supplementary material for: α-KG inhibits tumor growth of diffuse large B-cell lymphoma by inducing ROS and TP53-mediated ferroptosis
Source: Cell Death Discov. 2023 Jun 12;9:182. doi: 10.1038/s41420-023-01475-1 (PMC10260963; doi:10.1038/s41420-023-01475-1)
Supplement: Supplementary file 1 — Supplementary Figures [file 41420_2023_1475_MOESM1_ESM.docx]

**Supplementary Fig. S1 Expression analysis of glutamine metabolism and serum α-KG.**

**
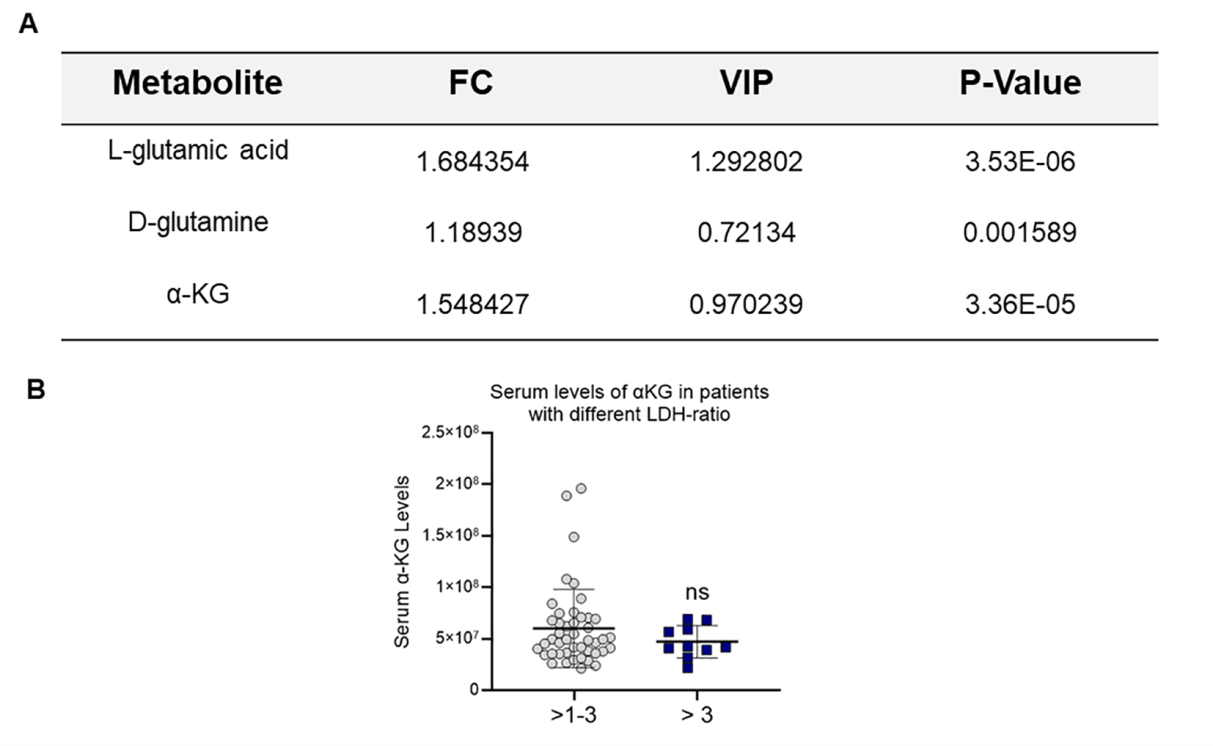
**

**A.** Fold change (FC), variance importance (VIP) and p-value of L-glutamic acid, D-glutamine, and α-KG in glutamine metabolism. **B.** DLBCL patients were categorized into two groups with LDH-ratio>1-3 and LDH-ratio>3, further comparing the levels of KG in two groups. The data were analyzed by two-tailed t-test. Ns=no significance.

**Supplementary Fig. S2 α-KG induced apoptosis in Val and U2932 cell lines.**


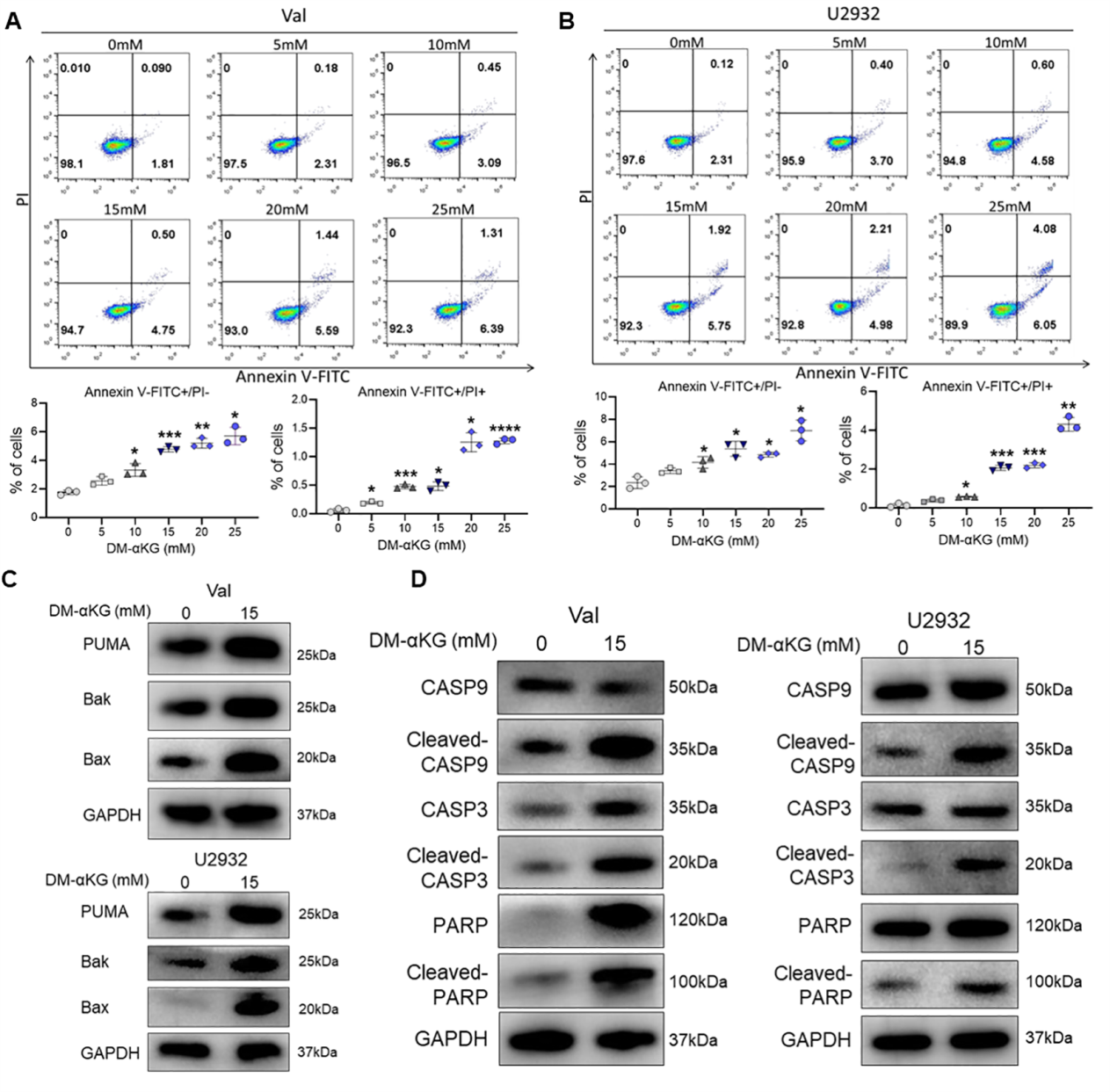


**A, B.** Val and U2932 cells were treated with different concentration of DM-αKG for 24h, followed by flow cytometry analysis. Representative flow cytometry plots, quantitative Annexin V-FITC+/PI-, and quantitative Annexin V-FITC+/PI+ were showed in Val (**A**) and U2932 (**B**). Data were presented as means ± SD of three independent experiments. The data were analyzed using one-way ANOVA followed by Dunnett’s multiple comparison tests. ^*^P<0.05, ^**^P<0.01, ^***^P<0.001, ^****^P<0.0001. **C, D.** Val and U2932 cells were treated with 0.1% DMSO and 15mM DM-αKG for 24h. Immunoblot analysis revealed the expression of apoptotic pathways.

**Supplementary Fig. S3 LDHB expression was decreased by DM-αKG treatment in DLBCL cell lines.**


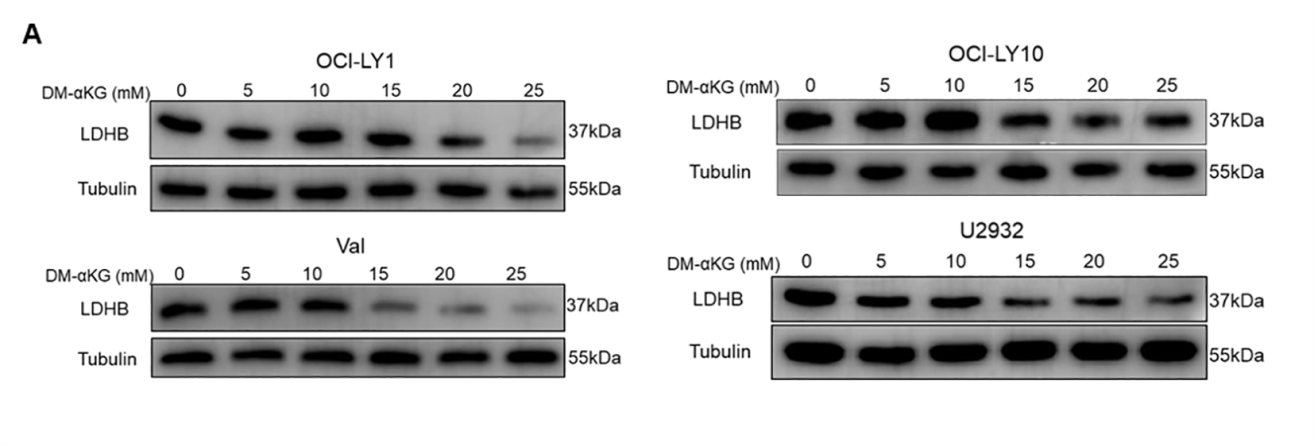


**A**. OCI-LY1, Val, OCI-LY10, and U2932 cell lines were treated with different concentrations of DM-αKG (0, 5, 10, 15, 20, 25mM) for 24h. The protein levels of LDHB were detected in DM-αKG-treated cells by immunoblot analysis.

**Supplementary Fig. S4 Differentially expressed genes (DEGs) in DM-αKG-treated cells.**

**
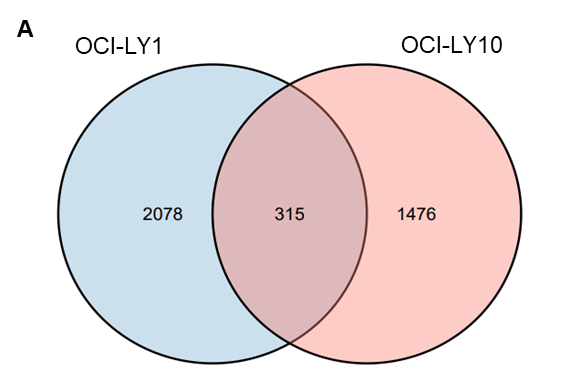
**

**A.** RNA-sequencing was performed on OCI-LY1 and OCI-LY10 cells treated with DM-αKG for 24 hours. Subsequently, DEGs were screened and displayed by Venn diagram. The results showed 2078 DEGs in OCI-LY1 and 1476 DEGs in OCI-LY10. In addition, 315 genes had intersections.

**Supplementary Fig. S5 Gene set enrichment analysis (GSEA) of DM-αKG-treated OCI-LY1 and OCI-LY10 cells.**


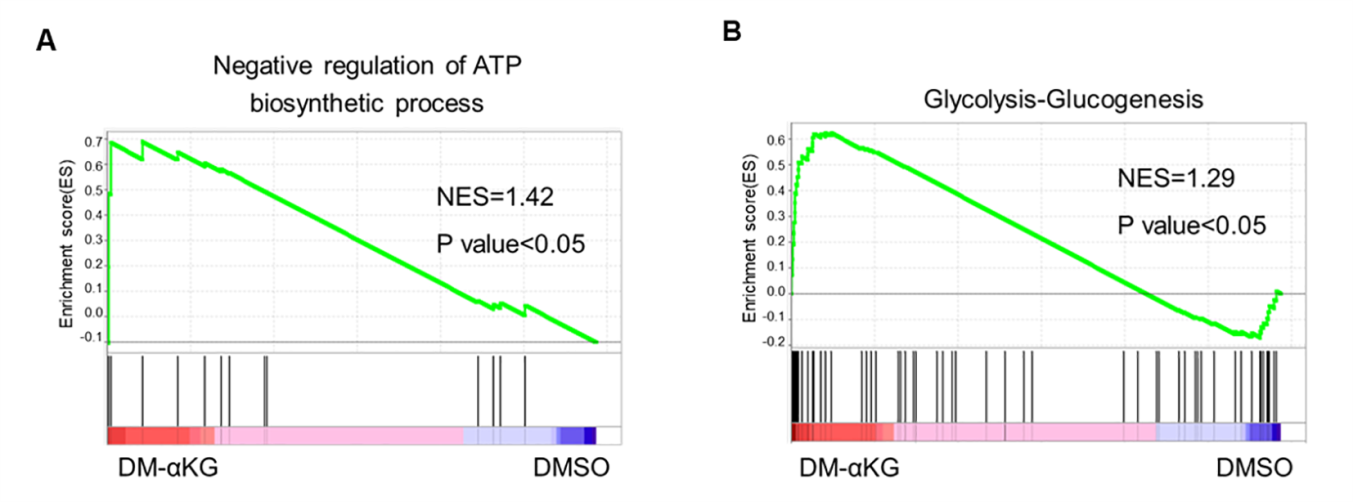


**A.** GSEA of negative regulation of ATP biosynthetic process (NES=1.42, p<0.05). **B.** GSEA of glycolysis-glucogenesis pathways (NES=1.29, p<0.05).
