# Supplementary material for: α-KG inhibits tumor growth of diffuse large B-cell lymphoma by inducing ROS and TP53-mediated ferroptosis
Source: Cell Death Discov. 2023 Jun 12;9:182. doi: 10.1038/s41420-023-01475-1 (PMC10260963; doi:10.1038/s41420-023-01475-1)
Supplement: Supplementary file 2 — Supplementary Tables [file 41420_2023_1475_MOESM2_ESM.docx]

**Supplementary Table S1.** **Treatment regimens of enrolled patients.**

| **Treatment regimens** | **Number of Patients (n=53)** |
| --- | --- |
| 3 cycles of R-CHOP | 11 |
| 6 cycles of R-CHOP | 12 |
| 8 cycles of R-CDOP+X (Orelabrutinib or Zanubrutinib) | 3 |
| 8 cycles of R-CHOP+Orelabrutinib | 3 |
| 4 cycles of R2-CHOP and 3 cycles of R-CHOP | 2 |
| Others | 22 |

1. R-CHOP: Rituximab, Cyclophosphamide, Doxorubicin, Vincristine and oral Prednisone.

2. R-CDOP: Rituximab, Cyclophosphamide, Liposomal doxorubicin, Vincristine and oral Prednisone.

3. R2-CHOP: Lenalidomide, Rituximab, Cyclophosphamide, Doxorubicin, Vincristine and oral Prednisone.

4. Others: Patients with more than 2 kinds of first-line regimens or second-line regimens. Second-line regimens included Cyclophosphamide + Doxorubicin + Vincristine + Prednisone + Etoposide (CHOPE), Methotrexate + Cytarabine (Hyper-CVAD B), local radiotherapy, allogeneic hematopoietic stem cell transplantation (ASCT), etc.

**Supplementary Table S2. Antibodies applied in immunoblot analysis.**

| **Antibodies** | **Resource** | **Dilution** |
| --- | --- | --- |
| Rabbit anti-p-ATM | Abcam, ab81292, Cambridge, MA, USA | 1:1000 |
| Rabbit anti-NRF1 | Abcam, ab175932, Cambridge, MA, USA | 1:1000 |
| Rabbit anti-LDHB | Abcam, ab53292, Cambridge, MA, USA | 1:1000 |
| Rabbit anti-p-Histone H2AX | Cell Signaling Technology, 9718, MA, USA | 1:1000 |
| Rabbit anti-TP53 | Cell Signaling Technology, 2527, MA, USA | 1:1000 |
| Rabbit anti-PUMA | Cell Signaling Technology, 98672, MA, USA | 1:1000 |
| Rabbit anti-Bak | Cell Signaling Technology, 12105, MA, USA | 1:1000 |
| Rabbit anti-Bax | Cell Signaling Technology, 5023, MA, USA | 1:1000 |
| Rabbit anti-CASP9 | Cell Signaling Technology, 9508, MA, USA | 1:1000 |
| Rabbit anti-cleaved-CASP9 | Cell Signaling Technology, 52873, MA, USA | 1:1000 |
| Rabbit anti-CASP3 | Cell Signaling Technology, 14220, MA, USA | 1:1000 |
| Rabbit anti-cleaved-CASP3 | Cell Signaling Technology, 9664, MA, USA | 1:1000 |
| Rabbit anti-PARP | Cell Signaling Technology, 9542, MA, USA | 1:1000 |
| Rabbit anti-cleaved-PARP | Cell Signaling Technology, 5625, MA, USA | 1:1000 |
| Rabbit anti-ATM | Proteintech, 27156-1-AP, IL, USA | 1:1000 |
| Mouse anti-PGC-1α | Proteintech, 66369-1-Ig, IL, USA | 1:2500 |
| Mouse anti-ESRRA | OriGene Technologies, TA808636S, MD, USA | 1:2000 |
| Mouse anti-GAPDH | Zhongshan Golden Bridge, TA-08, Beijing, China | 1:1000 |
| Mouse anti-Tubulin | Zhongshan Golden Bridge, TA-10, Beijing, China | 1:1000 |
| Anti-rabbit IgG, HRP-linked Antibody | Cell Signaling Technology, 7074, MA, USA | 1:5000 |
| Anti-mouse IgG, HRP-linked Antibody | Cell Signaling Technology, 7076, MA, USA | 1:5000 |
